# Supplementary material for: Sex, age, and racial/ethnic disparities in hyperuricemia prevalence and risk factors among U.S. adults: An analysis of NHANES 2007–2018 data
Source: PLoS One. 2026 Feb 25;21(2):e0337714. doi: 10.1371/journal.pone.0337714 (PMC12935233; doi:10.1371/journal.pone.0337714)
Supplement: S1 Table — Adjusted for age, race/ethnicity, education level, marriage, income, body mass index, hypertension, diabetes, renal function (eGFR), alcohol consumption, smoking status. (DOCX) [file pone.0337714.s001.docx]

Table S1. Univariate and Multivariate Logistic Regression Analysis of Risk Factors for Male Hyperuricemia Prevalence excluding gout participants (NHANES 2007–2018)

|  | Male Patients with Hyperuricemia/n(%) | Univariate OR (95%CI) | Multivariate OR (95%CI) | Univariate *p* | Multivariate *p* |
| --- | --- | --- | --- | --- | --- |
| **Age** |  |  |  |  |  |
| 20-29 | 537 (19.48%) | 1.00 (Referent) | 1.00 (Referent) |  |  |
| 30-39 | 540 (19.60%) | 1.01 (0.88 ,1.15) | 0.76 (0.66, 0.88) | 0.908 | < 0.001 |
| 40-49 | 473 (18.54%) | 0.94 (0.82 ,1.08) | 0.6 (0.52, 0.7) | 0.385 | < 0.001 |
| 50-59 | 469 (18.52%) | 0.94 (0.82 ,1.08) | 0.52 (0.44, 0.61) | 0.377 | < 0.001 |
| 60-69 | 489 (19.06%) | 0.97 (0.85 ,1.12) | 0.44 (0.37, 0.52) | 0.697 | < 0.001 |
| 70-79 | 313 (20.70%) | 1.08 (0.92 ,1.26) | 0.38 (0.31, 0.46) | 0.338 | < 0.001 |
| ≥80 | 211 (22.98%) | 1.23 (1.03 ,1.48) | 0.4 (0.32, 0.51) | 0.022 | < 0.001 |
| **Race/ethnicity, n (%)** |  |  |  |  |  |
| Non-Hispanic white | 1278 (20.04%) | 1.00 (Referent) | 1.00 (Referent) |  |  |
| Non-Hispanic black | 714 (21.79%) | 1.11 (1 ,1.23) | 1.19 (1.06, 1.33) | 0.045 | 0.003 |
| Mexican American | 383 (15.82%) | 0.75 (0.66 ,0.85) | 0.78 (0.68, 0.9) | < 0.001 | 0.001 |
| Others | 657 (18.69%) | 0.92 (0.83 ,1.02) | 1.15 (1.03, 1.29) | 0.104 | 0.014 |
| **Education** |  |  |  |  |  |
| Some high school | 740 (18.25%) | 1.00 (Referent) | 1.00 (Referent) |  |  |
| High school or GED | 751 (20.13%) | 1.13 (1.01 ,1.26) | 1 (0.89, 1.13) | 0.035 | 0.972 |
| Some college | 889 (21.21%) | 1.21 (1.08 ,1.34) | 1.03 (0.91, 1.17) | 0.001 | 0.616 |
| College graduate | 652 (18.05%) | 0.99 (0.88 ,1.11) | 0.95 (0.83, 1.08) | 0.818 | 0.42 |
| **Marital status, n (%)** |  |  |  |  |  |
| Married or living with a partner | 1888 (19.03%) | 1.00 (Referent) | 1.00 (Referent) |  |  |
| Living alone | 1144 (20.18%) | 1.08 (0.99 ,1.17) | 1.09 (0.99, 1.19) | 0.081 | 0.065 |
| **Ratio of family income to poverty** |  |  |  |  |  |
| ≤ 1.0 | 605 (19.08%) | 1.00 (Referent) | 1.00 (Referent) |  |  |
| 1.0 to 2.0 | 829 (19.49%) | 1.03 (0.91 ,1.15) | 0.95 (0.84, 1.07) | 0.659 | 0.385 |
| ＞ 2.0 | 1598 (19.57%) | 1.03 (0.93 ,1.15) | 0.99 (0.88, 1.13) | 0.554 | 0.936 |
| **Body mass index** |  |  |  |  |  |
| ≤ 24.9kg/m2 | 467 (10.23%) | 1.00 (Referent) | 1.00 (Referent) |  |  |
| 25.0 kg/m2 to 29.9kg/m2 | 1035 (17.85%) | 1.91 (1.7 ,2.14) | 1.89 (1.67, 2.13) | < 0.001 | < 0.001 |
| ≥ 30.0kg/m2 | 1530 (29.28%) | 3.63 (3.25 ,4.07) | 3.64 (3.23, 4.1) | < 0.001 | < 0.001 |
| **Alcohol Use** |  |  |  |  |  |
| No | 720 (18.21%) | 1.00 (Referent) | 1.00 (Referent) |  |  |
| Yes | 2312 (19.87%) | 1.11 (1.01 ,1.22) | 1.22 (1.11, 1.35) | 0.023 | < 0.001 |
| **Diabetes** |  |  |  |  |  |
| No | 2625 (19.29%) | 1.00 (Referent) | 1.00 (Referent) |  |  |
| Yes | 407 (20.50%) | 1.08 (0.96 ,1.21) | 0.78 (0.68, 0.89) | 0.203 | < 0.001 |
| **Hypercholesterolemia** |  |  |  |  |  |
| No | 1268 (22.13%) | 1.00 (Referent) | 1.00 (Referent) |  |  |
| Yes | 1764 (17.89%) | 0.77 (0.71 ,0.83) | 0.74 (0.68, 0.81) | < 0.001 | < 0.001 |
| **Hypertension** |  |  |  |  |  |
| No | 1720 (16.50%) | 1.00 (Referent) | 1.00 (Referent) |  |  |
| Yes | 1312 (25.39%) | 1.72 (1.59 ,1.87) | 1.53 (1.39, 1.68) | < 0.001 | < 0.001 |
| **Coronary heart disease** |  |  |  |  |  |
| No | 2846 (19.23%) | 1.00 (Referent) | 1.00 (Referent) |  |  |
| Yes | 186 (23.40%) | 1.28 (1.08 ,1.52) | 0.98 (0.81, 1.18) | 0.004 | 0.83 |
| **Glomerular filtration rate (GFR)** |  |  |  |  |  |
| GFR ≥ 90mL/min | 1477 (15.93%) | 1.00 (Referent) | 1.00 (Referent) |  |  |
| GFR 60 to 89mL/min | 1107 (21.50%) | 1.45 (1.33 ,1.58) | 1.87 (1.68, 2.08) | < 0.001 | < 0.001 |
| GFR 30 to 59mL/min | 400 (37.88%) | 3.22 (2.81 ,3.69) | 5.13 (4.29, 6.13) | < 0.001 | < 0.001 |
| GFR < 30mL/min | 48 (42.11%) | 3.84 (2.64 ,5.59) | 6 (3.98, 8.98) | < 0.001 | < 0.001 |

Adjusted for age, race/ethnicity, education level, marriage, income, body mass index, hypertension, diabetes, renal function (eGFR), alcohol consumption, smoking status.
