# Supplementary material for: Sex, age, and racial/ethnic disparities in hyperuricemia prevalence and risk factors among U.S. adults: An analysis of NHANES 2007–2018 data
Source: PLoS One. 2026 Feb 25;21(2):e0337714. doi: 10.1371/journal.pone.0337714 (PMC12935233; doi:10.1371/journal.pone.0337714)
Supplement: S2 Table — Adjusted for age, race/ethnicity, education level, marriage, income, body mass index, hypertension, diabetes, renal function (eGFR), alcohol consumption, smoking status. (DOCX) [file pone.0337714.s002.docx]

S2 Table. Univariate and Multivariate Logistic Regression Analysis of Risk Factors for Female Hyperuricemia Prevalence excluding gout participates (NHANES 2007–2018)

|  | Male Patients with Hyperuricemia/n(%) | Univariate OR (95%CI) | Multivariate OR (95%CI) | Univariate *p* | Multivariate *p* |
| --- | --- | --- | --- | --- | --- |
| **Age** |  |  |  |  |  |
| 20-29 | 234 (8.69%) | 1.00 (Referent) | 1.00 (Referent) |  |  |
| 30-39 | 246 (8.59%) | 0.99 (0.82, 1.19) | 0.76 (0.63, 0.93) | 0.894 | 0.006 |
| 40-49 | 310 (10.33%) | 1.21 (1.01, 1.45) | 0.7 (0.58, 0.85) | 0.036 | < 0.001 |
| 50-59 | 482 (17.62%) | 2.25 (1.9, 2.65) | 0.95 (0.79, 1.16) | < 0.001 | 0.63 |
| 60-69 | 643 (23.00%) | 3.14 (2.67, 3.68) | 0.97 (0.79, 1.18) | < 0.001 | 0.746 |
| 70-79 | 484 (28.72%) | 4.23 (3.57, 5.02) | 0.97 (0.78, 1.21) | < 0.001 | 0.788 |
| ≥80 | 339 (29.76%) | 4.45 (3.7, 5.35) | 0.95 (0.74, 1.22) | < 0.001 | 0.693 |
| **Race/ethnicity, n (%)** |  |  |  |  |  |
| Non-Hispanic white | 1136 (16.97%) | 1.00 (Referent) | 1.00 (Referent) |  |  |
| Non-Hispanic black | 803 (22.26%) | 1.4 (1.27, 1.55) | 1.42 (1.26, 1.6) | < 0.001 | < 0.001 |
| Mexican American | 279 (10.93%) | 0.6 (0.52, 0.69) | 0.76 (0.65, 0.89) | < 0.001 | 0.001 |
| Others | 520 (12.82%) | 0.72 (0.64, 0.8) | 1.11 (0.98, 1.27) | < 0.001 | 0.102 |
| **Education** |  |  |  |  |  |
| Some high school | 685 (17.01%) | 1.00 (Referent) | 1.00 (Referent) |  |  |
| High school or GED | 658 (17.94%) | 1.07 (0.95, 1.2) | 1.09 (0.95, 1.25) | 0.284 | 0.217 |
| Some college | 895 (16.84%) | 0.99 (0.89, 1.1) | 1.1 (0.97, 1.26) | 0.82 | 0.136 |
| College graduate | 500 (12.81%) | 0.72 (0.63, 0.81) | 1.08 (0.92, 1.26) | < 0.001 | 0.344 |
| **Marital status, n (%)** |  |  |  |  |  |
| Married or living with a partner | 1301 (14.34%) | 1.00 (Referent) | 1.00 (Referent) |  |  |
| Living alone | 1437 (18.34%) | 1.34 (1.24, 1.46) | 0.95 (0.86, 1.04) | < 0.001 | 0.272 |
| **Ratio of family income to poverty** |  |  |  |  |  |
| ≤ 1.0 | 655 (16.46%) | 1.00 (Referent) | 1.00 (Referent) |  |  |
| 1.0 to 2.0 | 811 (17.66%) | 1.09 (0.97, 1.22) | 1 (0.88, 1.13) | 0.141 | 0.981 |
| ＞ 2.0 | 1272 (15.25%) | 0.91 (0.82, 1.01) | 0.94 (0.83, 1.06) | 0.084 | 0.302 |
| **Body mass index** |  |  |  |  |  |
| ≤ 24.9kg/m2 | 339 (6.60%) | 1.00 (Referent) | 1.00 (Referent) |  |  |
| 25.0 kg/m2 to 29.9kg/m2 | 656 (13.55%) | 2.22 (1.93, 2.54) | 1.99 (1.72, 2.31) | < 0.001 | < 0.001 |
| ≥ 30.0kg/m2 | 1743 (25.13%) | 4.75 (4.2, 5.37) | 4.44 (3.89, 5.09) | < 0.001 | < 0.001 |
| **Alcohol Use** |  |  |  |  |  |
| No | 1355 (17.91%) | 1.00 (Referent) | 1.00 (Referent) |  |  |
| Yes | 1383 (14.80%) | 0.8 (0.73, 0.86) | 1.15 (1.04, 1.26) | < 0.001 | 0.005 |
| **Diabetes** |  |  |  |  |  |
| No | 2135 (14.34%) | 1.00 (Referent) | 1.00 (Referent) |  |  |
| Yes | 603 (29.79%) | 2.53 (2.28, 2.82) | 1.14 (1.01, 1.29) | < 0.001 | 0.039 |
| **Hypercholesterolemia** |  |  |  |  |  |
| No | 1246 (17.87%) | 1.00 (Referent) | 1.00 (Referent) |  |  |
| Yes | 1492 (15.01%) | 0.81 (0.75, 0.88) | 0.82 (0.75, 0.9) | < 0.001 | < 0.001 |
| **Hypertension** |  |  |  |  |  |
| No | 1019 (9.43%) | 1.00 (Referent) | 1.00 (Referent) |  |  |
| Yes | 1719 (28.15%) | 3.76 (3.46, 4.1) | 1.9 (1.72, 2.11) | < 0.001 | < 0.001 |
| **Coronary heart disease** |  |  |  |  |  |
| No | 2608 (15.81%) | 1.00 (Referent) | 1.00 (Referent) |  |  |
| Yes | 130 (31.18%) | 2.41 (1.95, 2.98) | 0.98 (0.77, 1.24) | < 0.001 | 0.853 |
| **Glomerular filtration rate (GFR)** |  |  |  |  |  |
| GFR ≥ 90mL/min | 971 (9.30%) | 1.00 (Referent) | 1.00 (Referent) |  |  |
| GFR 60 to 89mL/min | 1035 (20.68%) | 2.54 (2.31, 2.79) | 2.08 (1.85, 2.34) | < 0.001 | < 0.001 |
| GFR 30 to 59mL/min | 632 (47.77%) | 8.92 (7.86, 10.12) | 6.87 (5.8, 8.15) | < 0.001 | < 0.001 |
| GFR < 30mL/min | 100 (68.03%) | 20.74 (14.57, 29.52) | 15.5 (10.62, 22.93) | < 0.001 | < 0.001 |

Adjusted for age, race/ethnicity, education level, marriage, income, body mass index, hypertension, diabetes, renal function (eGFR), alcohol consumption, smoking status.
