# Supplementary material for: Sex, age, and racial/ethnic disparities in hyperuricemia prevalence and risk factors among U.S. adults: An analysis of NHANES 2007–2018 data
Source: PLoS One. 2026 Feb 25;21(2):e0337714. doi: 10.1371/journal.pone.0337714 (PMC12935233; doi:10.1371/journal.pone.0337714)
Supplement: Attachment — Submitted filename: Respones.to.Reviewers.5.docx [file pone.0337714.s004.docx]

**Dear Editor,**

We thank you and the reviewers for your careful evaluation of our manuscript, *“Sex, Age, and Racial/Ethnic Disparities in Hyperuricemia Prevalence and Risk Factors Among U.S. Adults: An Analysis of NHANES 2007–2018 Data.”* We appreciate all the comments and suggestions, which have helped us improve the clarity and quality of the paper. We have revised the manuscript accordingly. Below, we provide a point-by-point response to each comment raised by the editor and reviewers. For clarity, the original comments are quoted *in italics* (indented), and our responses follow. The corresponding revisions in the manuscript have been highlighted in red. A clean version of the revised manuscript has also been uploaded.

**Editor’s Comments**

**Comment 1 (Editor):** *“Is hyperuricemia solely hazardous? Hyperuricemia may be related to cardiovascular events, but the causal relationship is still ambiguous. I could not understand why the author underlined that targeted screening and interventions are essential for hyperuricemia.”*

**Response:**

Thank you very much for raising this insightful question, which gives us the opportunity to clarify further the rationale behind emphasizing “targeted screening and intervention for hyperuricemia (HUA)” in our manuscript. We fully acknowledge that the causal relationship between hyperuricemia and cardiovascular events remains uncertain, a viewpoint supported by several recent Mendelian randomization studies and prospective cohort investigations^1,2^.

It was not our intention to imply that hyperuricemia is invariably harmful across all populations, nor to advocate for universal intervention in the general public. Our emphasis on “targeted screening and intervention” stems from the fact that, even in the absence of definitive causal evidence, extensive epidemiological studies have consistently shown that hyperuricemia is associated with an increased risk of gout^3^, progression of chronic kidney disease^4^, metabolic abnormalities, and incident cardiovascular events^5^. Moreover, among individuals with established cardiometabolic or renal risk factors—such as hypertension, obesity, or chronic kidney disease—elevated uric acid often serves as an early and modifiable biomarker^6,7^.

Therefore, by “targeted screening and intervention,” we refer to strategies focused on specific high-risk groups—such as those with recurrent gout attacks, impaired renal function, or multiple metabolic risk factors. In these populations, timely monitoring and evidence-based intervention when clinically indicated may help reduce the incidence of complications. We have expanded on the scope and rationale of this approach in the Discussion section to avoid overgeneralizing our recommendations to the general population^8^.

We have revised the Conclusions(L507-L510) to state that targeted screening and interventions may be warranted in specific high-risk groups, rather than implying it is essential for everyone. These changes ensure a balanced discussion that does not overstate the hazards of hyperuricemia while still highlighting the public health relevance of our findings.

**Comment 2 (Editor):** *“Gout is caused by the precipitation of uric acid, which is derived from hyperuricemia. The relationship between gout and hyperuricemia is apparent, so there is no need to elaborate on it further.”*

**Response:**

We agree and have streamlined the manuscript to avoid over-elaborating on the well-known relationship between hyperuricemia and gout. In the Introduction, we initially provided background on gout as a consequence of hyperuricemia; we have now condensed this to a brief statement for context only. Any redundant or lengthy explanation of gout’s relationship to hyperuricemia has been removed. This revision keeps the focus on our study’s objectives and findings, without reiterating established knowledge.

**Comment 3 (Editor):** *“Did you consider medication use? The discrepancy in sex may be attributed to differences in medication use between the sexes, and you should discuss this further.”*

**Response:**

Thank you for raising this critical point. In the revised manuscript, we have gone beyond discussion and included an analysis of two classes of medications available in NHANES that directly affect serum uric acid levels: diuretics and urate-lowering therapy (ULT).

Our weighted regression models demonstrate that the use of diuretics (e.g., furosemide) was strongly associated with increased odds of hyperuricemia in both sexes. After full adjustment, the odds ratio for males was 2.67 (95% CI: 1.97–3.61) and for females was 2.55 (95% CI: 1.98–3.27). Conversely, the use of urate-lowering medications (e.g., allopurinol, febuxostat) was significantly associated with a reduced risk of hyperuricemia. The adjusted odds ratio was 0.57 (95% CI: 0.35–0.95) in males and 0.55 (95% CI: 0.21–1.43) in females, thou-gh the result for females did not reach statistical significance, likely due to limited sample size. These findings confirm the expected biological effects of these medications and highlight their role as essential covariates.

We also note sex-related differences: diuretic use was more common in older women (likely due to treatment of hypertension and heart failure), which may contribute to the higher prevalence of hyperuricemia observed in females after midlife. On the other hand, men had a higher prevalence of urate-lowering therapy use, reflecting their higher lifetime burden of gout, which may partly offset hyperuricemia prevalence in males.

In the revised Abstract (L78–82), Methods (L257–259), Results (L243–249), and Discussion (L422–433), we explicitly describe the findings on medication use and interpret them as contributing to the observed sex disparity in hyperuricemia prevalence. **With the inclusion of medication variables in the multivariable models (see Tables 2 and 3), estimates for other risk factors became more robust.** This addition strengthens the manuscript by moving beyond speculation and providing direct evidence of the impact of medication use on serum uric acid levels.

Table 2. Weighted Univariate and Multivariate Logistic Regression Analysis of Risk Factors for Hyperuricemia in Males (NHANES 2007–2018)

Adjusted for age, race/ethnicity, education level, marriage, income, body mass index, hypertension, diabetes, renal function (eGFR), alcohol consumption, smoking status, history of gout, diuretic use, and urate-lowering therapy use.

Table 3. Weighted Univariate and Multivariate Logistic Regression Analysis of Risk Factors for Hyperuricemia in Females (NHANES 2007–2018)

Adjusted for age, race/ethnicity, education level, marriage, income, body mass index, hypertension, diabetes, renal function (eGFR), alcohol consumption, smoking status, history of gout, diuretic use, and urate-lowering therapy use.

**Comment 4 (Editor):** *“Please only suggest the results and avoid adding interpretation in the Results section. For example, ‘…, indicating that obesity is one of the most important modifiable risk factors.’ in BMI. Such interpretation should be in the Discussion section.”*

**Response:** We have revised the Results section to remove any interpretative statements, ensuring it focuses strictly on the empirical findings. Specifically, we deleted the phrase “indicating that obesity is one of the most important modifiable risk factors” from the Results section where we report the association with BMI.

We relocated this interpretative insight to the Discussion(L414-L416) section, where we discuss the implications of the strong association between obesity and hyperuricemia. The revised manuscript is as followed:

“The association between obesity and hyperuricemia was robust, emphasizing obesity as a critical modifiable risk factor for hyperuricemia prevention strategies”.

Throughout the Results, we now present the data without commentary, and we reserve interpretation, context, and implications for the Discussion. This change aligns the manuscript with the journal’s guidelines and clearly separates results from interpretation.

**Comment 5 (Editor):** *“Typo: ‘based on the clinical guidelines….’ Please use the uppercase letters. Additionally, please add the reference to the definition of hyperuricemia (‘Hyperuricemia Identification’ in ‘Data Collection and Variable Definitions’).”*

**Response:**

Thank you very much for your careful review and thoughtful corrections regarding these details. We have made the following revisions based on your suggestions:

1) **Correction of Spelling and Formatting**
   In the “Data Collection and Variable Definitions” section, the phrase “based on the clinical guidelines” has been revised to “Based on the clinical guidelines,” with the initial letter capitalised in accordance with English writing conventions. We have also conducted a thorough review of the entire manuscript to ensure that similar spelling and capitalization issues have been uniformly corrected to maintain consistency in formatting.
2) **Addition of Reference for the Definition of Hyperuricemia**
   In the “Hyperuricemia Identification” subsection, we have supplemented the definition of hyperuricemia with an authoritative reference to allow readers to access the original source. The revised manuscript is as followed:
   “Hyperuricemia was defined as a serum uric acid (SUA) concentration >7.0 mg/dL (>416 μmol/L) in males and >6.0 mg/dL (>357 μmol/L) in females. These thresholds were based on the 2018 European Alliance of Associations for Rheumatology (EULAR) evidence-based recommendations for the diagnosis of gout^7^ and the 2020 American College of Rheumatology (ACR) guideline for the management of gout^9^”

**Comment 6 (Editor):** *“I highly recommend using English editing by native.”*

**Response:**

We have thoroughly revised the manuscript for English language and style. A native English-speaking colleague (and professional editor) assisted in copyediting the text. We corrected grammatical errors, improved sentence clarity, and eliminated awkward phrasing. The overall readability of the manuscript has been significantly improved. Additionally, we took this opportunity to ensure that the formatting and style conform to PLOS ONE guidelines. For example, we adjusted the manuscript to meet the journal’s formatting requirements for headings, figures, and references. We trust that the language and presentation of the paper now meet the high standards expected by the journal.

**Reviewer #1’s Comments**

**Comment (Reviewer 1):** *“Hello. This is a good article, and it explores an important topic. It seems that more studies are needed in this field in the future to be able to state with certainty the impact of exposure to such toxic substances. Good luck.”*

**Response:**

We thank Reviewer #1 for the positive feedback and encouragement. We are pleased that the importance of the topic was acknowledged. We agree that further studies (especially longitudinal and interventional research) are needed to conclusively determine the causal impacts of chronic hyperuricemia on health outcomes. In the revised Discussion, we have noted that our cross-sectional findings highlight associations and disparities that warrant prospective investigation. We also emphasize in the Conclusion that continued research will be important to confirm and extend our findings. We appreciate the reviewer’s insight and have kept this perspective in mind, indicating that while our study adds to the knowledge in this field, establishing causal effects will require future investigations.

**Reviewer #2’s Comments**

**Comment 1 (Reviewer 2):** *“Consider the issue of reverse causality: Hyperuricemia is a precursor condition to gout, as the authors also acknowledge in the introduction. Including ‘history of gout’ as a covariate in the multivariable regression model may introduce reverse causality bias. I recommend conducting a sensitivity analysis excluding individuals with a history of gout to better assess the risk factors for hyperuricemia.”*

**Response:**

We sincerely appreciate this valuable suggestion regarding potential reverse causality. We have carefully addressed this concern through a sensitivity analysis, and the key revisions are as follows:

1) **Sensitivity analysis**:
   As recommended, we conducted a sensitivity analysis by excluding all participants with a self-reported history of gout and re-ran the multivariable logistic regression models for hyperuricemia.
2) **Summary of Findings**:
   The results from this analysis remained highly consistent with those from the primary model. Specifically:
   - No material changes were observed in the significance or direction of any key risk factors.
   - For instance, in females, the adjusted odds ratio for obesity changed only marginally from 4.29 (full sample) to 4.44 (gout-excluded sample), with fully overlapping 95% confidence intervals.
   - Similar stability was noted across all sex and racial/ethnic subgroups.
   - 3) **Manuscript Additions**:
   - A statement has been added to the Results section highlighting the consistency of the sensitivity analysis results.
   - Two new supplementary tables (**Table S1** for males and **Table S2** for females) have been included, presenting adjusted prevalence and odds ratios after excluding participants with gout.
   - 4) **Interpretation**:
     The minimal effect of excluding gout patients suggests that reverse causality did not substantially bias our original estimates. This reinforces the robustness of the identified risk factors for hyperuricemia, even after accounting for potential behavioral or pharmacological modifications among gout patients.
5) **Discussion Update**:
   We have added a comment in the Discussion noting that the associations reported are robust to the exclusion of individuals with a history of gout.

Table S1. Univariate and Multivariate Logistic Regression Analysis of Risk Factors for Male Hyperuricemia Prevalence excluding gout participants (NHANES 2007–2018)

Table S2. Univariate and Multivariate Logistic Regression Analysis of Risk Factors for Female Hyperuricemia Prevalence excluding gout participants (NHANES 2007–2018)

Adjusted for age, race/ethnicity, education level, marriage, income, body mass index, hypertension, diabetes, renal function (eGFR), alcohol consumption, smoking status.

**Comment 2 (Reviewer 2):** *“Clarify laboratory methods: Please provide a detailed description of how serum uric acid was measured in NHANES, including the assay method, equipment, and quality control procedures if available. This will improve reproducibility and methodological transparency.”*

**Response:** Thank you for your suggestions. We have expanded the Methods section to include a more detailed description of the laboratory measurement of serum uric acid in NHANES.

In the revised “Laboratory Measurements” subsection(L196-L211), we now state that serum uric acid was measured enzymatically using a uricase-based method. We specify the instruments used by NHANES: in the 2007–2008 cycle, uric acid was measured with a Beckman Synchron LX20 auto-analyzer, and from 2008 onward NHANES utilized the Beckman Coulter UniCel DxC800 analyzer. The assay involved a timed endpoint method in which uricase catalyzes the oxidation of uric acid to allantoin and hydrogen peroxide; the reaction is coupled to a colorimetric change that is measured photometrically, and the change in absorbance is directly proportional to the uric acid concentration in the sample.

We also note that NHANES employed rigorous quality control procedures: all laboratory testing followed standardized protocols with regular calibration, and quality control samples were analyzed to ensure accuracy and consistency. NHANES laboratory data are subject to external quality assurance through the Centers for Disease Control and Prevention’s protocols. These details have been added to the Methods for transparency. We believe that providing this information will help with reproducibility and give readers confidence in the reliability of the laboratory data.

**Comment 3 (Reviewer 2):** *“Definition of hyperuricemia: Specify clearly which guideline was used to define hyperuricemia (e.g., ACR, EULAR, or another standard). Please cite the source and briefly discuss the clinical rationale for the chosen cut-offs.”*

**Responses:**

Thank you very much for the constructive feedback. In the revised manuscript, we have provided a detailed explanation regarding the source and clinical rationale for the definition of hyperuricemia used in this study(L178-L194).

In this study, hyperuricemia was defined as a serum uric acid level >7.0 mg/dL (>416 μmol/L) in men and >6.0 mg/dL (>357 μmol/L) in women. These thresholds were established with reference to the 2018 European League Against Rheumatism (EULAR) evidence-based recommendations for the diagnosis of gout^7^, the 2020 American College of Rheumatology (ACR) Guideline for the Management of Gout^9^.

The clinical rationale for selecting these cut-offs is based on the following aspects:

1. **Urate Solubility Theory**: Under physiological temperature and pH conditions, serum uric acid concentrations exceeding approximately 7 mg/dL can lead to the precipitation and deposition of monosodium urate crystals in joints and tissues, thereby increasing the risk of gout^12^.
2. **Sex-Specific Differences**: Due to the uricosuric effect of estrogen, premenopausal women generally exhibit lower serum uric acid levels than men. Therefore, a lower threshold for women (6.0 mg/dL) is commonly applied in both clinical and epidemiological contexts to improve the detection sensitivity of hyperuricemia^7,10^.
3. **International Comparability**: These diagnostic cut-offs are widely used in major population studies, including NHANES, facilitating valid comparisons with previous research findings^11,12^.

**Comment 4 (Reviewer 2):** *“Provide more detail on covariates: Describe key variables more clearly. For example, what were the specific categories of education? Were these self-reported during interviews, or collected through another method (e.g., online forms or medical examination)?”*

**Response:**

Thank you very much for the reviewer’s attention to the details of covariate definitions and for their valuable suggestions. We agree that clearly describing the categorization and data sources of key variables is essential to help readers understand the methodology and enhance the reproducibility of the results.

In the revised manuscript, we have expanded the “**Definition of Covariates**” subsection within the Methods section to provide detailed definitions and categorization for key sociodemographic and clinical variables. For example:

- **Education level** was categorized into: “Less than high school”, “High school graduate/“General Educational Development (GED) ”, “Some college or an Associate of Arts (AA) degree”, and “College graduate or above”.
- **Data source**: Education information was self-reported during the NHANES household interview.

We have also clarified the definitions and data collection methods for other key covariates. The following details have been added to the **Methods (L196-L240)**:

A range of potential covariates was evaluated based on the existing literature^13^, encompassing age, sex, marital status, race/ethnicity, education level, family income, gout, hypercholesterolemia, hypertension, diabetes, coronary heart disease, alcohol consumption, BMI, and GFR. Race/ethnicity was stratified into non-Hispanic White, non-Hispanic Black, Mexican American, and other ethnic groups. Marital status was categorised as married or living alone. Education level was divided into less than high school, high school graduate or GED, some college or associate degree, and college graduate or above. Family income was classified into three groups based on the poverty income ratio (PIR): low (PIR ≤ 10), medium (PIR > 1 to 2.0), and high (PIR > 3). A history of gout， hypertension, diabetes, and coronary heart disease was determined based on self-reported physician-diagnosed conditions. Hypercholesterolemia was defined as a total serum cholesterol concentration ≥240 mg/dL^14^, consistent with the National Cholesterol Education Program Adult Treatment Panel III (NCEP ATP III) criteria and widely used in NHANES-based epidemiological studies. Total cholesterol was measured from blood samples obtained in the Mobile Examination Center (MEC) and analyzed in accordance with standardized laboratory protocols. Alcohol drinking status was determined using the survey question “In any 1 year, have you had at least 12 drinks of any type of alcoholic beverage?” Participants who answered “yes” were classified as alcohol drinkers. Body mass index (BMI) was calculated as weight in kilograms divided by height in meters squared (kg/m²), based on standardized anthropometric measurements collected during the MEC physical examination. Estimated glomerular filtration rate (eGFR) was calculated using the Chronic Kidney Disease Epidemiology Collaboration (CKD-EPI) creatinine equation, based on serum creatinine measured in MEC laboratory assessments.

**Comment 5.1 (Reviewer 2):** *“Specify which statistical software and packages were used.”*

**Response:** We have now included the specific software and packages used for our analysis in the Methods section (L252-L255). All analyses were conducted using R (version 4.2.2). We utilized the **survey** package in R to properly account for the complex NHANES sampling design during -our statistical computations. We also used standard R libraries for data management and analysis (for example, the **dplyr** package for data manipulation and base R **stats** functions for regression modeling). By specifying the software (R) and key packages, we align with reproducibility standards and address the reviewer’s request.

**Comment 5.2 (Reviewer 2):** *“Confirm whether survey weights were applied during analysis, given the complex NHANES sampling design.”*

**Response:** We appreciate this important comment. In the original submission, our logistic regression models did **not** incorporate the NHANES survey weights. Following your suggestion, in the revised manuscript we have re-analyzed all data using the appropriate complex survey design procedures(L253-L257).

Specifically, we applied the 2-year MEC examination weights (WTMEC2YR), divided by 6 to create a 12-year pooled weight (since six NHANES cycles from 2007–2018 were combined), and incorporated strata (SDMVSTRA) and primary sampling units (SDMVPSU) as recommended in NHANES analytic guidelines. Analyses were performed in R (version 4.2.2) using the survey package (functions svydesign and svyglm), which correctly accounts for the complex sampling design.

After re-analysis, we found that the main risk factors for hyperuricemia (e.g., obesity, hypertension, impaired renal function) remained robustly significant, with odds ratios of similar magnitude to those in the unweighted analysis. However, a few associations showed changes: for example, in females, the association with alcohol use became non-significant after weighting, while the association with diabetes became significant. These results highlight the importance of using survey weights to ensure nationally representative estimates.

In line with this update, **Tables 2 and 3** in the revised manuscript have been replaced with the weighted results, and their footnotes now explicitly state that weights, strata, and PSUs were incorporated. We believe this revision strengthens the rigor and validity of our findings.

**Comment 5.3 (Reviewer 2):** *“Provide more information about how covariates were selected for inclusion in the multivariable models (e.g., based on prior literature, significance in univariable models, directed acyclic graphs, etc.).”*

**Response:** We have expanded the explanation of our covariate selection strategy in the Statistical Analysis section.

1) In the revised text, we note that covariates were chosen based on a combination of **prior literature**^11,13,15^ and empirical considerations. Before building the multivariable model, we identified variables known to be associated with hyperuricemia or gout from previous studies (such as age, sex, race/ethnicity, obesity, hypertension, kidney function, alcohol use, etc.). We decided *a priori* to include these key factors due to their established relevance.
2) Additionally, we examined univariable **logistic regression** in our data; any potential risk factor that showed a meaningful association with hyperuricemia (e.g., p < 0.20 in univariable analysis) was considered for inclusion in the multivariable model.
3) We avoided including **highly collinear variables** together (for example, we included body mass index as the measure of adiposity rather than including both BMI and waist circumference in the same model, since those are strongly correlated). Our final multivariable model thus included the major demographic and clinical factors that were justified by literature and our data. We have stated this rationale in the manuscript so that readers understand how we arrived at the set of covariates in the model (Table 2). This addresses the reviewer’s concern about transparency in model building.

**Comment 5.4 (Reviewer 2):** *“Indicate whether regression diagnostic tests (such as checking for multicollinearity, goodness-of-fit, or influential observations) were conducted.”*

**Response:** We have now added a description of the diagnostic checks we performed for our logistic regression models.

1) In the Statistical Analysis section, we mention that we assessed multicollinearity among the independent variables by calculating variance inflation factors (VIF). All VIF values were below 2, indicating that multicollinearity was not a concern in our models.
2) We also evaluated the goodness-of-fit of the main logistic regression model for hyperuricemia. For instance, we performed the Hosmer-Lemeshow test, which yielded a p-value of 0.45, suggesting no evidence of poor fit.
3) Additionally, we examined influential observations using standardized residuals and leverage statistics; no single data point had an unduly large influence on the model estimates. We include a sentence in the revised manuscript noting that these diagnostic checks were conducted and that no major violations of model assumptions were detected. By documenting these steps, we aim to reassure readers (and the reviewer) that our findings are robust and not an artifact of model misspecification or outliers.

**Comment 6 (Reviewer 2):** *“Contextualize findings with previous NHANES data: It would strengthen the discussion to mention hyperuricemia prevalence from prior NHANES cycles (e.g., 1988–1994, 1999–2000) and highlight any trends over time. This would help situate the current findings within the broader epidemiological context.”*

**Response:** We agree that placing our results in historical context enriches the discussion. In response, we have added a new paragraph in the Discussion section that explicitly compares our results (2007–2018) with data from previous NHANES surveys. The added content outlines the secular trends in hyperuricemia prevalence among U.S. adults over the past three decades:

- During the NHANES III (1988–1994) period, the estimated prevalence of hyperuricemia was approximately 19.1%. This figure increased to around 19.6% during the 1999–2000 cycle^16^.
- A further rise was observed in 2007–2008, with prevalence reaching 21.5%^10^, which aligns closely with the overall prevalence reported in our study (21.6% in 2017–2018).
- More recent data from the 2015–2016 cycle indicate that hyperuricemia remains highly prevalent, affecting approximately 20.1% of U.S. adults^11^.

This persistent upward trend, particularly evident since the late 1980s, likely reflects concurrent increases in major hyperuricemia risk factors such as obesity and hypertension over the same period.

We discuss how our study (2007–2018) fits into this context: our observed overall prevalence (21.6% in 2017–2018 in our data) and the demographic patterns are consistent with the continuing burden of hyperuricemia. Furthermore, we highlight that our finding of post-menopausal women eventually surpassing men in hyperuricemia prevalence mirrors observations from past data that showed the sex gap narrowing in older age groups. By adding these comparisons, we provide readers with a clearer picture that hyperuricemia has been an increasing (and now persistent) public health issue over the last several decades. This broader epidemiological context underscores the importance of targeted prevention efforts today, as recommended by our study.

Once again, we thank the reviewers and the editor for their thorough and constructive feedback. We believe the revisions have substantially improved the manuscript. We have addressed all the points raised, and we are happy to make any further changes if needed. We hope that the revised manuscript now meets the requirements for publication in PLOS ONE.

Sincerely,

Shengguang-Li

**Reference**

1.	White, J. *et al.* Plasma urate concentration and risk of coronary heart disease: a mendelian randomisation analysis. *Lancet, Diabetes Endocrinol.* **4**, 327–336 (2016).

2.	Li, X. *et al.* Serum uric acid levels and multiple health outcomes: umbrella review of evidence from observational studies, randomised controlled trials, and Mendelian randomisation studies. *BMJ* **357**, j2376 (2017).

3.	Kuo, C.-F., Grainge, M. J., Mallen, C., Zhang, W. & Doherty, M. Rising burden of gout in the UK but continuing suboptimal management: a nationwide population study. *Ann. Rheum. Dis.* **74**, 661–667 (2015).

4.	Li, L. *et al.* Is hyperuricemia an independent risk factor for new-onset chronic kidney disease?: a systematic review and meta-analysis based on observational cohort studies. *BMC Nephrol.* **15**, 122 (2014).

5.	Feig, D. I., Kang, D.-H. & Johnson, R. J. Uric acid and cardiovascular risk. *N Engl J Med* **359**, 1811–1821 (2008).

6.	Kuwabara, M. *et al.* Asymptomatic hyperuricemia without comorbidities predicts cardiometabolic diseases: five-year japanese cohort study. *Hypertens. (dallas Tex,: 1979)* **69**, 1036–1044 (2017).

7.	Richette, P. *et al.* 2018 updated european league against rheumatism evidence-based recommendations for the diagnosis of gout. *Ann. Rheum. Dis.* **79**, 31–38 (2020).

8.	Brucato, A., Cianci, F. & Carnovale, C. Management of hyperuricemia in asymptomatic patients: a critical appraisal. *Eur. J. Intern. Med.* **74**, 8–17 (2020).

9.	FitzGerald, J. D. *et al.* 2020 American College of Rheumatology Guideline for the Management of Gout. *Arthritis Care Res (Hoboken)* **72**, 744–760 (2020).

10.	Zhu, Y., Pandya, B. J. & Choi, H. K. Comorbidities of gout and hyperuricemia in the US general population: NHANES 2007-2008. *Am. J. Med.* **125**, 679-687.e1 (2012).

11.	Chen‐Xu, M., Yokose, C., Rai, S. K., Pillinger, M. H. & Choi, H. K. Contemporary Prevalence of Gout and Hyperuricemia in the United States and Decadal Trends: The National Health and Nutrition Examination Survey, 2007–2016. *Arthritis Rheumatol.* **71**, 991–999 (2019).

12.	Dalbeth, N., Gosling, A. L., Gaffo, A. & Abhishek, A. Gout. *Lancet (lond. Engl.)* **397**, 1843–1855 (2021).

13.	Zou, Y., Liu, Y. & Li, S. Association between dietary vitamin C intake and gout among american adults. *Front. Immunol.* **15**, 1431323 (2024).

14.	National Cholesterol Education Program (NCEP) Expert Panel on Detection, Evaluation, and Treatment of High Blood Cholesterol in Adults (Adult Treatment Panel III). Third report of the national cholesterol education program (NCEP) expert panel on detection, evaluation, and treatment of high blood cholesterol in adults (adult treatment panel III) final report. *Circulation* **106**, 3143–3421 (2002).

15.	Zou, Y. *et al.* Associations of weight change patterns with hyperuricemia risk in U.S. adults. *Sci. Rep.* **15**, 21124 (2025).

16.	Prevalence of hyperuricemia and gout and their relationship to certain risk factors: a systematic review. *Researchgate* doi:10.36106/ijar/5312074.
